# Effects of Boric Acid Ester Modified Magnesium Borate Whisker on the Mechanical Properties and Crystallization Kinetics of Polypropylene Composites

**DOI:** 10.3390/ma13071698

**Published:** 2020-04-05

**Authors:** Jin-Hua Luo, Shi-Hu Han, Juan Wang, Hui Liu, Xiao-Dong Zhu, Shan-Hua Chen

**Affiliations:** 1 College of Vanadium and Titanium, Panzhihua University, Panzhihua 617000, China; ljhazq020312@163.com; 2 College of Materials and Chemistry & Chemical Engineering, Chengdu University of Technology, Chengdu 610059, China; herry13@126.com; 3 Eternal Chemical Co., Ltd., Chengdu 611535, China; 4 College of Mechanical Engineering, Chengdu University, Chengdu 610106, China; wangjuan@stu.cdu.edu.cn (J.W.); L2976505495@163.com (H.L.)

**Keywords:** polypropylene, magnesium borate whisker, composite, mechanical properties, non-isothermal crystallization behavior

## Abstract

Polypropylene (PP) is notch sensitive and brittle under severe conditions of deformation, limiting wider range of its usage as a structural load-bearing polymer. Hence, in this work the magnesium borate whisker (MBw), with similar mechanical properties to carbon fiber but much less expensive than polycrystalline silicon carbide, was modified by boric acid ester (BAE) and then used to fabricate PP composites. The mechanical properties, morphology, and non-isothermal crystallization property of virgin PP, PP/MBw, and PP/BAE-MBw composites were studied through mechanical testing, scanning electron microscopy (SEM), and differential scanning calorimetry (DSC), respectively. The non-isothermal crystallization data was analyzed via Mo, Kissinger, and Dobreva methods. The results reveal that the incorporation of BAE-MBw into PP matrix results in higher tensile strength and impact strength than those of virgin PP and PP/MBw composite. The activation energies based on Kissinger were 190.20 kJ/mol for virgin PP, 206.59 kJ/mol for PP/MBw, and 218.98 kJ/mol for PP/BAE-MBw. The nucleation activities of whiskers determined by the Dobreva model were 0.86 for PP/MBw and 0.75 for PP/BAE-MBw. As a result, the whiskers, especially the modified whiskers, act as active substrates to facilitate heterogeneous nucleation, which leads to an increase in crystallization rate.

## 1. Introduction

Polypropylene (PP) has been widely used as the major polymeric material in daily necessities, construction, and packaging, and within the automobile industry and for other industrial applications, owing to its exceptional comprehensive properties, low manufacturing cost, and ease of processing [1,2,3]. However, the low mechanical properties at low temperature and the notch-sensitive feature could hinder the wider application of PP [4,5,6]. Therefore, in order to achieve desired property combinations which are difficult to obtain from virgin PP, embedding various fillers (particulates, fibers, layered inorganic clays and whiskers, etc.) in PP has become a main method [7]. Owing to a near-perfect crystal structure and high tensile strength, fibrous whiskers, such as silicon carbide, aluminum borate, and potassium titanate, are particularly attractive for increasing the mechanical, thermal, and dielectric properties of polymers [8,9,10,11,12,13,14,15,16]. Among them, magnesium borate whisker (MBw) has attracted much attention due to its low thermal expansion coefficient and high Young’s modulus, as well as cheapness [17].

To obtain PP/MBw composite with good performance, processes should be developed which could promote the interfacial adhesion between constituent materials, because the apparent difference in the surface property between nonpolar hydrophobic PP and hydrophilic MBw could result in poor interfacial interaction [18]. For instance, MBw is prone to aggregate, and often exhibits poor dispersion in the PP matrix, which could deteriorate the tensile strength of PP. Thus, it is necessary to convert the hydrophilic surface of the MBw into a hydrophobic one, in order to improve the interfacial affinity between PP and MBw [19]. This is typically accomplished through surface modification of MBw using coupling agents capable of reacting with hydroxyl groups. Herein, coupling agents used for surface modification may also have a remarkable impact on PP crystallization, and thereby on the mechanical properties of the PP/MBw composites. It is well known that fillers in the composites play a role as nucleation agent to form nucleating sites, leading to a faster crystallization rate of polymer, and a higher crystallization temperature [20]. Likewise, it is also important in this research to understand the effects of whisker on PP crystallization behavior.

In this paper, a boric acid ester (BAE) was used to modify MBw for the purpose of reducing agglomeration and enhancing the interfacial adhesion between MBw and PP matrix. Firstly, the PP/MBw and PP/BAE-MBw composites were prepared via one-step melt blending in one pot. Subsequently, the mechanical performances and fracture morphologies of all samples were studied by means of tensile and impact testing, and SEM, respectively. Finally, the non-isothermal crystallization kinetics of all samples was investigated via differential scanning calorimetry (DSC) test and Mo method. The activation energy and nucleation activities of MBw and BAE-MBw in PP matrix were also calculated based on the Kissinger and Dobreva methods, respectively.

## 2. Experimental

### 2.1. Materials

PP (T30S) with melt flow index, M_W_, polydispersity index, isotacticity, impact strength, Rockwell hardness, flexural modulus, tensile yield strength, density, and Vicat softening point of 3.88 g (10 min)^−1^ (230 °C, 2.16 kg), about 3.99 × 10^5^ g/mol, ca. 4.6, 95.0%–96.5%, 6.66 kJ/m^2^, 90R, 1218 MPa, 34.76 MPa, 0.899 g/cm^3^, 153 °C, respectively, was provided from Sinopec Maoming Petrochemical Company, China. The BAE coupling agent and MBw are the same in our previous research [21]. High purity deionized water made by ourselves was used as solvent.

### 2.2. Composites Preparation

The preparation and analysis of BAE coupling agent modified MBw were also detailed in our previous research [21]. First, the raw materials were processed in a vacuum oven under the condition of 100 °C for 12 h. Then, PP composites containing 2, 5, 10 and 15 wt.% MBw and BAE-MBw were respectively prepared by melt mixing on a TDGC−2/0.5 two-roll mixing mill (Shanghai electric set factory Co., Ltd., Shanghai, China) at 190 °C with a rotor speed of 60 rpm for 8 min. Subsequently, the resultant composites were molded into plates using a HP−63 (D) hot-press machine (Shanghai Zimmerli Weili Co., Ltd., Shanghai, China) at 185 °C with a pressure of 10 MPa for 5 min. The molded plates were cooled in the hot-press under the same pressure for 6 min, and then prepared into standard samples for mechanical tests after 48 h.

### 2.3. Mechanical Property Testing

The tensile strength was measured on a Model 5567 30 kN load Frame Testing Unit (Instron, Norwood, MA, USA) with a tensile speed of 50 mm/min according to ISO 527-1:1993 standards, and the stress-strain data were automatically recorded by the testing system in the tensile process. Determination of Izod impact strength was performed on V-notched specimen (length l = 80 ± 2 mm, width b = 10.0 ± 0.2, thickness h = 4.0 ± 0.2 and tip radius 0.25 ± 0.05) using an impact tester (XJU-22, Beijing AVIC Times Equipment Co. Ltd., Beijing, China), according to ISO 180/A:2000 standards. The test parameters were speed 3.5 m/s, pendulum energy 11 J, pendulum tilt angle 160°, rounded cutting edge radius R = 0.8 + 0.2 mm, jaw fillet radius R = 1 + 0.1mm, impact of the blade angle 30 ± 1°, pendulum impact of air 0.5%. All the tests were done at room temperature, and the values reported reflected an average from five tests. The fracture morphologies of PP composites were obtained using scanning electron microscope (SEM, JSM7500F, JEOL Ltd., Tokyo, Japan). To obtain the real content of the filler in the SEM images, a commercial image processing tool (Image Pro-Plus 6, Media Cybernetics, Inc., Rockville, MD, USA) was used to analyze the average distance between the incorporated fillers in the composites. Additional details about the experiment are available elsewhere [18].

### 2.4. DSC Tests

The non-isothermal crystallization behaviors were investigated on a thermal analysis apparatus (TA, Q20, New Castle, DE, USA) under nitrogen. About 6–8 mg of samples of PP and PP composites containing 15 wt.% whiskers were placed in a corundum crucible and heated to 210 °C (10 °C/min), then held for 5 min in order to remove prior thermal history. Subsequently, the melt was cooled to 40 °C under a selection of constant cooling rates (*Φ*) varying from 5–30 °C/min. Relative degree of crystallinity *X(t)* as a function of crystallization temperature *T_t_* was calculated by using the recorded DSC curves of each sample according to Equation (1):

(1) $$X\left(t\right)=\frac{\int_{T_{0}}^{T_{t}}\left(dH_{c}/dT\right)dT}{\int_{T_{0}}^{T_{\infty }}\left(dH_{c}/dT\right)dT}$$

where *dH_c_* is crystallization enthalpy, and *T_0_*, *T_t_*, and *T_∞_* represent the onset temperature, temperature at time *t*, and temperature at end of crystallization process. The crystallization time *t* was calculated by Equation (2):

(2) $$t=\frac{T_{0}-T_{t}}{\Phi }$$

The half time of non-isothermal crystallization (*t*_1/2_) was calculated by the following relationship:

(3) $$t_{1/2}=\frac{T_{0}-T_{1/2}}{\Phi }$$

A new method proposed by Mo [22], based on Avrami and the Ozawa [23,24,25,26,27], is suitable for studying the non-isothermal crystallization curve as follow:

(4) logΦ=logF(T)−αlogt

Here, *F(T)* is a kinetic parameter, and *α* = Avrami exponent/Ozawa exponent. Higher *F(T)* means a higher cooling rate needed within unit crystallization time. The plot of log*Φ* against log *t* is a straight line, and the values of *F*(*T*) and *α* for PP, PP/MBw, and PP/BAE-MBw at different relative crystallinities were obtained from the plot intercept and plot slope, respectively.

Kissinger [28] developed a method to calculate the activation energy (*ΔE*) of the non-isothermal crystallization process as follows:

(5) $$d[\ln(\Phi /T_{p}^{2})]/d(1/Tp)=-\Delta E/R$$

in which *ΔE* represents the effective activation energy, *Tp* represents the peak temperature of crystallization, and *R* represents the ideal gas constant.

The nucleation activity (*ϕ)* of the dispersed whisker in the PP matrix was calculated following the Dobreva and Gutzow method [29,30]. For homogeneous nucleation from the PP melt, *Φ* is a function of the supercooling degree (Δ*T_p_* = *T_m_* − *T_p_*, *T_m_* is the melting temperature), i.e.,

(6) $$\log\Phi =A-\frac{B}{2.3\Delta T_{p}^{2}}$$

The *B* and constant *A* can be calculated based on the linear fitting curve determined by the Equation (6). However, when the system is heterogeneous nucleation, *B* needs to be replaced by B*. The nucleation activity *ϕ* = B*/*B*. *B* is defined by the following equation:

(7) $$B=\frac{\omega \sigma^{3}V_{m}^{2}}{3nkT_{m}\Delta S_{m}^{2}n}$$

where ω, σ, $V_{m}$, *n*, $\Delta S_{m}$, and *k* represent geometrical factor, specific energy, molar volume of the crystallizing substance, Avrami exponent, entropy of melting, and Boltzmann constant, respectively [31].

## 3. Results and Discussion

### 3.1. Mechanical Properties

**Figure 1:**
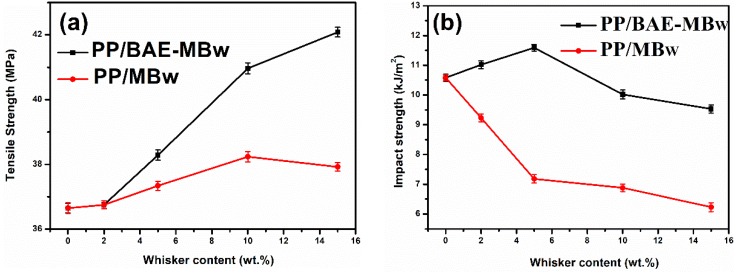
The variation of (**a**) tensile strength at yield and (**b**) impact strength with whisker contents for polypropylene (PP) composites.

**Figure 2:**
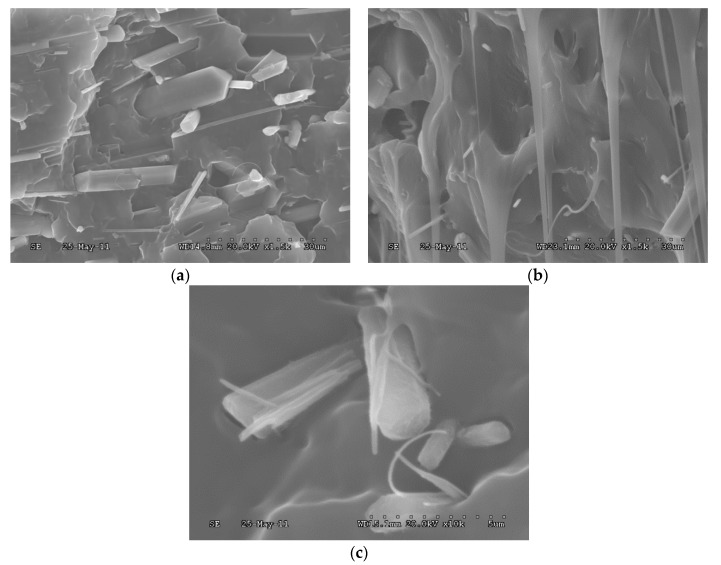
SEM images of the impact fracture surface of (**a**) PP/magnesium borate whisker (MBw), (**b**) PP/boric acid ester (BAE)-MBw (low magnification), and (**c**) PP/BAE-MBw (high magnification).

The change of mechanical properties of samples is displayed in Figure 1. It can be seen that the tensile strength of the PP/BAE-MBw increase with the increase in whisker content, but the impact strength decreases when the whisker content exceeds 5%. However, the above mechanical properties of PP/BAE-MBw are better than those of PP and PP/MBw. For the improvement of tensile strength, this may be because the addition of whisker facilitates load transmitting, and the presence of BAE coupling agent improves the efficiency of load transmitting. The reduction of impact strength, however, is a typical characteristic of polymer composites; that is, the macroscopic cracks are extremely easy to initiate and grow under impact test conditions. To further explore these results, the fracture morphologies of PP composites after impact test were observed through SEM. From Figure 2a, it is found that lots of unmodified whiskers are pulled out and appear to be debonded from the PP matrix. However, PP/BAE-MBw presents a rough transverse surface, and the whiskers are locked by PP well. These results indicate that BAE coupling agent can supply an intense interfacial adhesion between whisker and PP matrix, which is able to restrict the crack initiation and propagation rate, and therefore result in better mechanical properties for PP/BAE-MBw (Figure 2b,c) [14,32,33,34].

### 3.2. Crystallization Behavior

**Figure 3:**
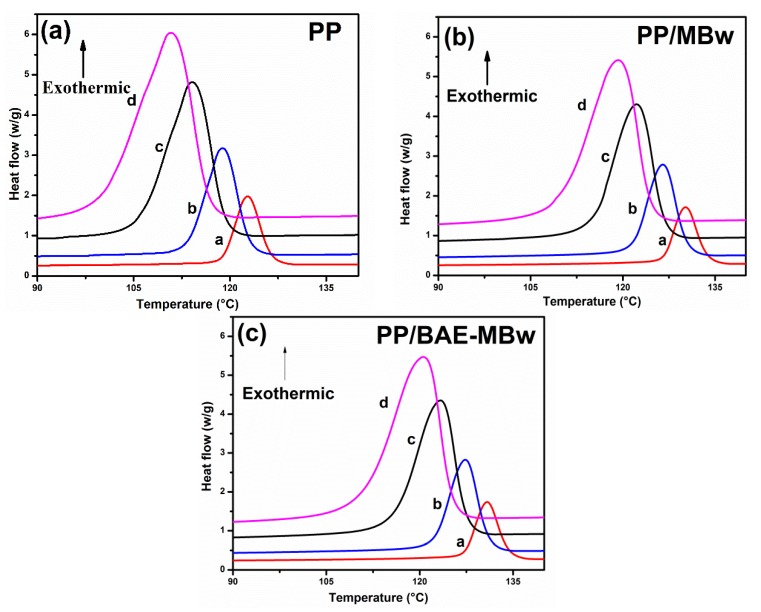
Differential scanning calorimetry (DSC) thermograms of non-isothermal crystallization of (**a**) PP, (**b**) PP/MBw, and (**c**) PP/BAE-MBw composites (cooling rate: a—5 °C/min, b—10 °C/min, c—20 °C/min, d—30 °C/min).

The tensile strength of the PP composite is still increased at 15 wt.% whiskers loading, indicating that whiskers may be not agglomerate in PP matrix, and maintain active nucleation. Therefore, it is meaningful to study the non-isothermal crystallization behavior of whiskers at this concentration. The non-isothermal crystallization exothermic curves of samples recorded from the melt state to 40 °C at different cooling rates are shown in Figure 3. The kinetic data*T_0_*, *T_p_*, and *∆H_C_* are listed in Table 1. The exothermic curve broadens and moves toward lower temperatures as cooling rate increases, and the *T_p_* of PP composites is enhanced significantly when MBw or BAE-MBw is added. This is because PP molecular chains have enough time to pass through the nucleation energy barrier on the condition of slow cooling rate, which will cause the crystallization to occur at a higher temperature, thereby increasing the *T_p_* value. When the cooling rate is fast, PP is easily crystallized at the supercooling temperature, since PP molecular chains cannot insert the crystal grains [35,36,37]. Under the same cooling rate, such as at a cooling rate of 5 °C/min, the *T_p_* values of 130.21 °C for PP/MBw and 130.83 °C for the PP/BAE-MBw are higher than 122.80 °C for the virgin PP, suggesting that the whiskers act as workable nucleating agents to decrease the degree of supercooling required for crystallization, which is similar to previous reports [38]. The value of *ΔH_C_* is reduced in the presence of whiskers, because the MBw destroy the integrity of PP matrix and reduce its activity, resulting in fewer crystallites in the PP composites. In addition, the higher *T_0_* and lower (*T_0_*−*T_p_*) of PP composites also indicate that the MBw and BAE-MBw could promote the crystallization of PP matrix. Furthermore, the nucleation ability of BAE-MBw is higher than MBw, probably due to a more uniform distribution of BAE-MBw than MBw in PP matrix [39,40,41].

### 3.3. Non-Isothermal Crystallization Analysis

**Figure 4:**
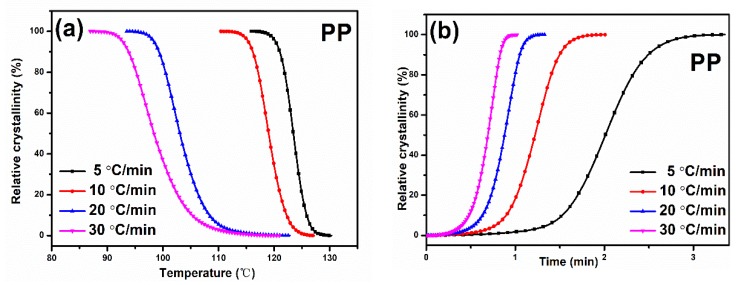
Representative curves of relative crystallinity versus (**a**) temperature and (**b**) time for virgin PP.

*X(t)* versus *T* and *t* are calculated by Equations (1) and (2). The representative curves and values of *t_1/2_* are shown in Figure 4 and Table 1, respectively. It is found that the higher the cooling rate, the less time required for completing crystallization, and *t_1/2_* values decrease as the cooling rate increases for all the samples. For a fixed cooling rate, PP shows a higher *t_1/2_* than PP composites. Thus, the incorporation of whisker in PP matrix can accelerate the overall non-isothermal crystallization process.

**Figure 5:**
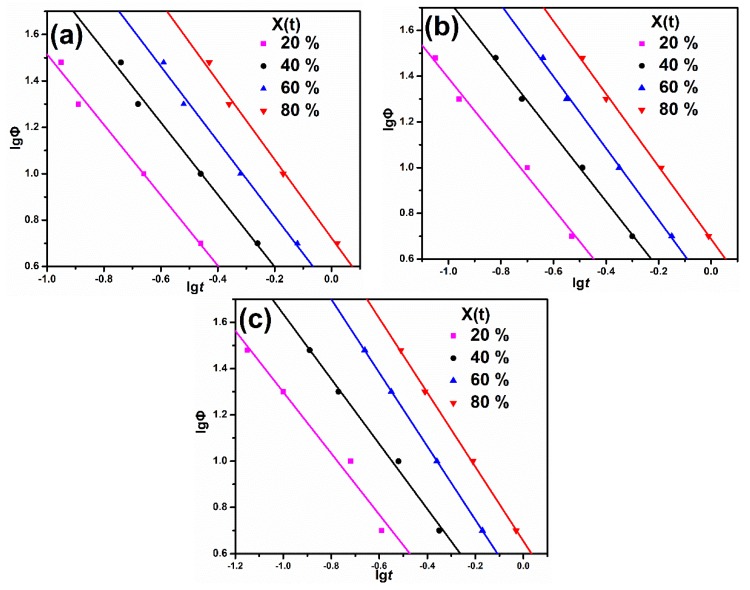
Plots of lg*Φ* versus lg*t* for PP and its composites at different relative degrees of crystallinity. (**a**) PP, (**b**) PP/MBw, and (**c**) PP/BAE-MBw.

Based on the Mo method (Equation (4)), the plots of log*Φ* versus log*t* for each sample at a given degree of crystallinity are shown in Figure 5. The α and *F(T)* values shown in Table 2 are obtained from the line intercepts and slopes in Figure 5. Obviously, Mo’s method successfully described the non-isothermal crystallization of all samples. The range of α values are 1.52−1.69 for the virgin PP, 1.53−1.59 for the PP/MBw, and 1.32−1.61 for the PP/BAE-MBw, suggesting the ratio of *n* to *m* for all samples almost remained constant at different crystallinities [42]. At a certain relative crystallinity, the *F(T)* value reflects the difficulty of the crystallization process, because the higher *F(T)* value means that a higher cooling rate is required to reach a certain crystallinity degree in a unit of time [43]. Table 2 exhibits the *F(T)* values. The *F(T)* value increases significantly with the increasing relative crystallinity, indicating that a higher cooling rate should be used for a given crystallization time to obtain a higher crystallinity. It is worth noting that the *F(T)* of PP is the highest and the PP/BAE-MBw is the lowest at the same relative crystallinity, revealing that PP/BAE-MBw exhibits the fastest crystallization rate. This result is consistent with the statement made from values of *t_1/2_*.

**Figure 6:**
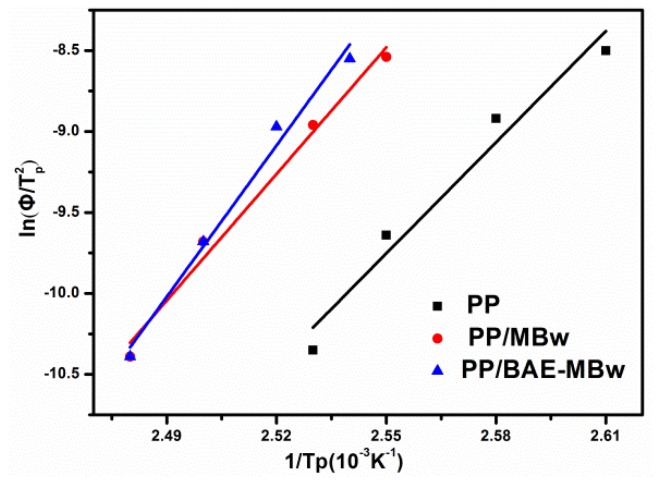
Plots of ln($\Phi /T_{p}^{2}$) versus 1/T_p_ for PP and PP composites.

Figure 6 presents the plots of ln($\Phi /T_{p}^{2}$) against 1/*T_p_* for PP and its composites, based on the Kissinger approach (Equation (5)). Table 2 exhibits the activation energy (Δ*E*) obtained from the plot’s slope. The Δ*E* values of PP, PP/MBw, and PP/BAE-MBw are 190.20, 206.59 and 218.98 kJ/mol. The Δ*E* values of PP composites are slightly larger than those of virgin PP. This may be due to the restriction of the movement of the PP molecular chain by whiskers, which results in an enhancement in the viscosity of the mixed melt [39,44]. The addition of MBw bring an increase in Δ*E*, however, the composites exhibit an enhancement in crystallization rate and a decline in the degree of subcooling required for crystallization, as shown in Figure 3, which may be caused by the nucleation of MBw.

**Figure 7:**
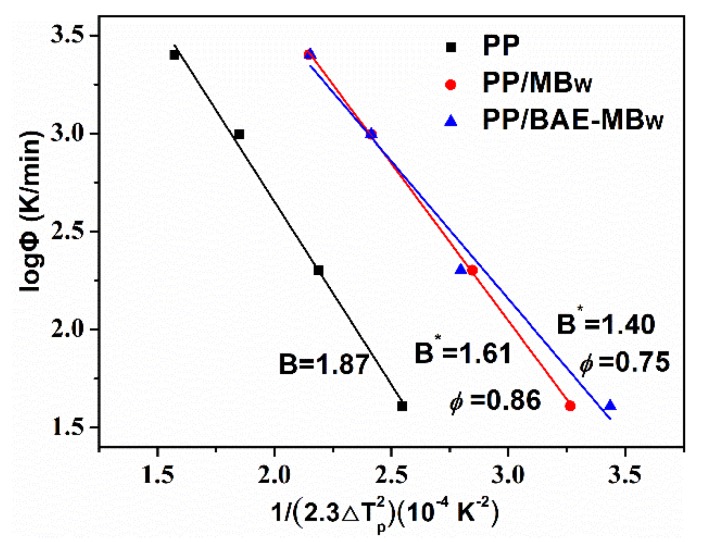
Plots of log*Φ* vs. 1/(2.3*∆*$T_{p}^{2}$) for PP and PP composites.

Figure 7 plots the log*Φ* versus 1/(2.3Δ$T_{p}^{2}$) for the virgin PP, PP/MBw, and PP/BAE-MBw. It is found that the *ϕ* value decreases with increasing MBw content. Thus, the MBw is an effective nucleating agent for PP matrix, and exhibits high nucleation activity in the hybrid melts.

## 4. Conclusions

1. The tensile strength and impact strength of PP/BAE-MBw are better than those of PP/MBw, which may be due to the more compatible interface between BAE-MBw and PP matrix.
2. The Mo and Kissinger models were found to describe the non-isothermal crystallization kinetics of PP, PP/MBw, and PP/BAE-MBw fairly well. The range of α values for virgin PP, PP/MBw, and PP/BAE-MBw are 1.52−1.69, 1.53−1.59, and 1.32−1.61, respectively. The crystallization rate of PP/BAE-MBw is the fastest among virgin PP, PP/MBw, and PP/BAE-MBw, which is in line with the values of *t_1/2_*. The Kissinger activation energy of PP, PP/MBw, and PP/BAE-MBw was calculated as 190.20 kJ/mol, 216.59 kJ/mol, and 258.98 kJ/mol, respectively. Thus, the MBw, especially for BAE-MBw, is an effective nucleating agent for PP matrix, and exhibits high nucleation activity in the hybrid melts.

## Figures and Tables

**Table 1:** Basic crystallization parameters of PP and its composites at different cooling rates.

Sample	*Φ*/°C∙min^−1^	*∆H_C_*/J∙g^−1^	*T_0_*/°C	*T_P_*/°C	(*T_0_ − T_P_*)/°C	*t*_1/2_/min	*T_m_*/°C
PP	5	102.17	126.43	122.80	3.63	2.01	164.12
	10	99.51	123.07	118.90	4.17	1.22	163.46
	20	98.70	119.23	114.15	5.08	0.88	162.64
	30	95.48	116.64	110.79	5.85	0.69	163.37
PP/MBw	5	85.98	133.73	130.21	3.52	1.52	165.78
	10	82.91	130.39	126.49	3.90	1.16	165.92
	20	80.17	126.77	122.17	4.60	0.84	164.61
	30	77.46	124.45	119.24	5.21	0.71	164.18
PP/BAE-MBw	5	87.42	134.30	130.83	3.47	1.38	167.32
	10	86.45	131.05	127.34	3.71	0.99	166.42
	20	81.89	127.48	123.32	4.16	0.83	165.76
	30	78.71	125.18	120.49	4.69	0.61	165.49

**Table 2:** Values of *α* and *F(T)* for PP and its composites at different relative degrees of crystallinity.

Sample	*X(t)*/%	*α*	*F(T)*	Δ*E*/kJ·mol^−1^
PP	20	1.52	0.99	190.20
	40	1.56	1.95	
	60	1.61	3.16	
	80	1.69	5.28	
PP/MBw	20	1.43	0.91	206.59
	40	1.47	1.84	
	60	1.57	2.86	
	80	1.59	4.79	
PP/BAE-MBw	20	1.32	0.92	218.98
	40	1.40	1.70	
	60	1.59	2.68	
	80	1.61	4.47	
